# Alterations in cellular pharmacokinetics and pharmacodynamics of elvitegravir in response to ethanol exposure in HIV-1 infected monocytic (U1) cells

**DOI:** 10.1371/journal.pone.0172628

**Published:** 2017-02-23

**Authors:** Narasimha M. Midde, Namita Sinha, Pradeep B. Lukka, Bernd Meibohm, Santosh Kumar

**Affiliations:** Pharmaceutical Sciences, College of Pharmacy, University of Tennessee Health Science Center, Memphis TN, United States of America; George Mason University, UNITED STATES

## Abstract

Ethanol consumption is negatively associated with antiretroviral therapy (ART) adherence and general health in HIV positive individuals. Previously, we demonstrated ethanol-mediated alterations to metabolism of elvitegravir (EVG) in human liver microsomes. In the current study, we investigated ethanol influence on the pharmacokinetic and pharmacodynamic interactions of EVG in HIV infected monocytic (U1) cells. U1 cells were treated with 5 μM EVG, 2 μM Cobicistat (COBI), a booster drug, and 20 mM ethanol for up to 24 hours. EVG, HIV p24 levels, alterations in cytochrome P450 (CYP) 3A4, MRP1, and MDR1 protein expressions were measured. Presence of ethanol demonstrated a significant effect on the total exposures of both EVG and EVG in combination with COBI. Ethanol also increased the HIV replication despite the presence of drugs and this elevated HIV replication was reduced in the presence of MRP1 and MDR1 inhibitors. Consequently, a slight increase in EVG concentration was observed in the presence of MRP1 inhibitor but not with MDR1 inhibitor. Furthermore, CYP3A4, MRP1 and MDR1 protein levels were significantly induced in treatment groups which included ethanol compared to those with no treatment. In summary, these findings suggest that Ethanol reduces intra cellular EVG exposure by modifying drug metabolism and transporter protein expression. This study provides valuable evidence for further investigation of ethanol effects on the intracellular concentration of EVG in ex vivo or in vivo studies.

## Introduction

Highly active antiretroviral therapy (HAART) regimen consisting of integrase strand transfer inhibitors (INSTI), nucleoside (or nucleotide) reverse transcriptase inhibitors (NRTI), nonnucleoside reverse transcriptase inhibitors (NNRTI), and protease inhibitors (PI) dramatically transformed HIV infection from fatal to a chronic but manageable disease. Among different classes of drugs, INSTIs such as elvitegravir (EVG), dolutegravir or raltegravir have become ‘standard’ drugs in the recommended regimens owing their superior efficacy and safety in clinical trials and retrospective evaluations[1, 2]. While highly effective, a major limitation of HAART therapy is its failure to eradicate the virus from cells even after years of therapy. Intracellular presence of EVG is a requisite for its action on its pharmacological target[3], while HIV therapeutics are optimized based on plasma pharmacokinetics[4]. Owing to most HAART therapeutic intracellular targets, it is of utmost importance to understand the intracellular pharmacokinetics of these essential drugs. This was further substantiated by several clinical studies which demonstrated a weak or no correlation of plasma INSTI, NRTIs or PIs concentration with antiviral efficacy in the treatment of HIV infected patients[5–8].

Intracellular EVG concentration is influenced by several factors including physicochemical properties of the drug, pharmacokinetic properties such as protein binding, hepatic metabolism, drug transporters and drug-drug interactions. Since EVG is predominantly metabolized by cytochrome P450 (CYP) 3A4[9] a strong CYP3A4 inhibitor such as cobicistat (COBI) is co-administered as a booster to increase EVG bioavailability. It was reported that EVG is a weak inducer of CYP3A4 and this effect can be countered by the presence of COBI in the liver[10]. But how EVG-drug interactions change the CYP3A4 induction at the cellular level has not been established. The intracellular antiretroviral drug concentrations are also dictated by the activity of efflux transporters[11, 12]. Several recent studies revealed that antiretroviral drugs can act as substrates and inducers for membrane transporters especially for efflux transporters[13, 14], which explain, at least in part, the reported high intracellular drug variability in HIV positive patients[15, 16]. Most of the recommended HAART therapy consists of multiple drugs and many of these drugs are known to modulate transporters. Moreover, concurrent use of other prescribed medication or drugs of abuse increases the complexity of the problem.

Ethanol, the most commonly consumed legal drugs in the world, is at low levels primarily metabolized by alcohol dehydrogenase (ADH), but at higher alcohol concentration both ADH and CYP2E1 are requires for metabolism[17]. Previously, we reported that exposure of ethanol increases the expression of both multidrug resistance protein 1 (MRP1) and CYP enzymes in U937 macrophages[18, 19]. Additionally, we reported altered CYP2E1 and CYP3A4 mRNA expression levels in chronic treatment of both ethanol and HIV protease inhibitors darunavir and ritonavir in macrophages[20]. At the systemic level, ethanol consumption (0.7 g/kg) with 600 mg of abacavir, an NRTI used in the treatment of HIV, increases abacavir total exposure by 41%, maximum concentration by 15% and half-life by 26% in 25 male HIV-infected individuals[21]. However, the effect of ethanol on the intracellular HIV drug concentration or efficacy in the clinical setting is not documented to date.

Since the intracellular concentrations of an active drug moiety determines the virologic suppression achieved in the body, understanding the drug interactions that lead to fluctuations in the intracellular drug concentrations are critical to improve treatment outcomes. However, there is a substantial knowledge gap on the impact of ethanol and HIV drug interactions at the cellular level, especially in monocytic cells. Monocytes are important targets of HIV-1 infection, and ART concentrations, especially PIs, is suboptimal in monocytes[22]. Further, HIV-infected monocytes infiltrate into the brain and spread the virus in perivascular macrophages and microglia that eventually form sanctuary sites for HIV-1 in the brain[23, 24]. In this study we examined the effect of acute exposure of ethanol and potential mechanism on the intracellular pharmacokinetics of EVG and its effectiveness on the viral suppression in HIV infected monocytic (U1) cells.

## Materials and methods

### Cell culture and treatments

HIV-infected U937 cell line—the U1 cells were obtained through the NIH AIDS Reagent Program (Germantown, MD). U1 cells were cultured in Roswell Park Memorial Institute (RPMI) 1640 media (Corning Inc, Tewksbury, MA) supplemented with 10% fetal bovine serum (Atlanta biologicals, Atlanta, GA), 1% L-glutamine, nonessential amino acid solution, sodium bicarbonate, and penicillin-streptomycin solution. Cells were plated at 0.9x10^6^ cells/well of 6-well plates and were differentiated into macrophages with 100 nM Phorbol 12-myristate 13-acetate (PMA) for 72 hours prior to treatment. On the day of experiment, medium was removed and cells were washed twice with PBS and treated with media containing clinically relevant concentrations of (a) EVG (5 μM), (b) EVG+COBI (2 μM), (c) EVG+ethanol (20 mM), (d) EVG+COBI+ethanol. Ethanol-treated plates were placed in an incubator that humidified with 20 mM ethanol to prevent any loss of ethanol due to evaporation.

At predetermined time points (0.5, 1, 3, 6, 12, and 24 hours), cells were collected for concentration-time profile experiments and medium was collected for HIV p24 measurement. At each time point, cells were washed twice with PBS and incubated with 400 μL of cold Radioimmunoprecipitation assay (RIPA) buffer for 10 minutes and the cell lysates were collected. For evaluation of the influence of efflux transporters, cells were incubated with either EVG+COBI+ethanol and MK-571 (MRP1 inhibitor) at 50 μM or PSC-833 (multidrug resistance1 (MDR1)) inhibitor at 5 μM for 24 hours before collecting cell lysate and media samples.

### LC-MS/MS analysis of elvitegravir

A previously reported LC-MS/MS method was used to quantitate EVG in cell lysates, culture medium, and plasma samples used in this study[25]. Calibrants (0.003, 0.006, 0.013, 0.025, 0.05, 0.1, 0.15, 0.25, 0.5, and 1 μM) were prepared by serial dilution approach with the corresponding working solutions. EVG extraction from the calibrants and study samples was performed by adding 3-volumes of cold acetonitrile which contained a 138 nM ritonavir (RTV) as internal standard (IS). All samples were centrifuged at 8000 rpm for 10 minutes at 4°C and clear supernatants were transferred into vails with low-volume inserts for LC-MS/MS analysis. Samples (5 μL) were injected onto a Waters Xterra® MS C18 Column (125Å, 3.5 μ, 4.6 X 50 mm) (Waters Corporation, Milford, MA) attached to a Shimadzu Nexera HPLC system coupled with a tandem mass spectrometer (Triple Quad 5500 from AB SCIEX, Framingham, MA) with electron spray ionization in positive mode. Data was acquired and analyzed with the Analyst software package (ver.1.6.2, AB SCIEX, Framingham, MA). Concentrations were quantified by the Quantitative Analysis module (Analyst ver 1.6.2, AB SCIEX, Framingham, MA) from a standard curve fitted with a linear-regression model with a weighting factor of 1/x^2^.

### HIV p24 ELISA

Influence of the combination of drugs on HIV replication was determined by measurement of HIV p24 levels in the cell culture medium. After initial screening of p24 levels at different time points, we decided to use the 24 hour samples from all the experiments for p24 quantitation which provided consistent optical density (OD) values. p24 antigen production was detected per manufacturer’s protocol (ZeptoMetrix Corp, Buffalo, NY). The concentration of p24 in the samples was quantitated by comparing with standards’ absorbance values and reported in percentage of control. The vehicle-treated samples were used as control and set as 100%.

### In-Cell Western analysis

Detecting low levels of protein expression in native cells using traditional Western blotting can be difficult and therefore some studies successfully used In-Cell Western blotting (ICW) to quantify minor alterations in protein expression[26, 27]. We used manufacturer recommended (LI-COR Biosciences, NE) protocol in this study to perform ICW. U1 cells (0.03x10^6^) were plated in sterile 96-well plates and differentiated with 100 nM PMA for 72 hours at 37°C in a humidified incubator while maintaining 5% CO_2_ air flow. On the day of treatment, each well was flushed with sterile PBS (250 μl) followed by addition of a 250 μl RPMI media containing various combinations of drugs previously mentioned. These plates were incubated for 24 hours prior to fixation. Medium from each well was removed by careful aspiration and cells were immediately fixed by addition of cold (-20°C) methanol (200 μl) followed by incubation of the plate at ambient temperature for 10 minutes with gentle shaking. Following incubation, methanol was carefully removed and wells were gently rinsed thrice with PBS (200 μl). After the last rinse, blocking was done by adding a 150 μl of Li-Cor blocking buffer and incubating the plate at room temperature with gentle shaking for 1.5 hours. Meanwhile, the primary antibodies were diluted in Li-Cor Odyssey blocking buffer using following dilutions: 3A4 (Rabbit Pab, Abcam # ab3572) 1: 200; MRP1 (mouse mab, Millipore # MAB4100) 1:50; MDR1(mouse mab, Millipore # MAB4334) 1:50; β-Actin (Mouse mAb Cell Signaling # 3700) 1:2000; β-Actin (Rabbit mAb Cell Signaling # 4970) 1:200. After blocking, 50 μl of desired antibody added to test wells and incubated overnight at 4°C with gentle shaking. On the following day, wells were washed five times with 200 μl PBS containing Tween-20 (0.1%) for 5 minutes at room temperature with gentle shaking. Secondary antibodies, goat anti-Mouse Mab (Li-Cor # 926–68170) and goat anti-Rabbit Mab (Li-Cor # 827–08365) were diluted in 1:1000 ratio in blocking buffer (0.2% -Tween-20, 0.01% SDS). After the final wash, wells were incubated with 50 μl of secondary antibody mixture for 1 hour in the dark at room temperature. After five washes, plates were scanned using Li-Cor Odyssey® SA and analyzed by using Image Studio V4.0 software (LI-COR Biosciences, NE). Background was subtracted from control wells and target protein signal intensities from triplicate wells were normalized to their respective β-actin intensities. Signal intensities were expressed as percent of relative responses (mean ± standard errors of mean) compared to vehicle controls.

### Pharmacokinetic and statistical analysis

The concentration-time profiles were analyzed by non-compartmental modeling using Phoenix WinNonlin 6.4 (Certara L.P., Princeton, NJ). Area under the curve (AUC_tot_) was calculated by the log trapezoidal rule by log-linear regression. The maximum concentration achieved (C_max_) and the time to maximum concentration (T_max_) were determined from the concentration-time profiles. Effect of ethanol on EVG AUC_tot_ and C_max_ were compared using unpaired t test with Welch's correction. T_max_ values were compared using Wilcoxon rank-sum test. All other data involving more than two groups were compared by one way ANOVA and multiple comparisons were performed by Tukey’s post hoc test. ANOVA data was presented along with multiplicity adjusted p values for each comparison. All the statistical analyses were done by Prism 6 (GraphPad Software, La Jolla, CA). A p-value ≤ 0.05 among treatment groups was considered significant.

## Results

### Intracellular ethanol-drug interaction

**Fig 1:**
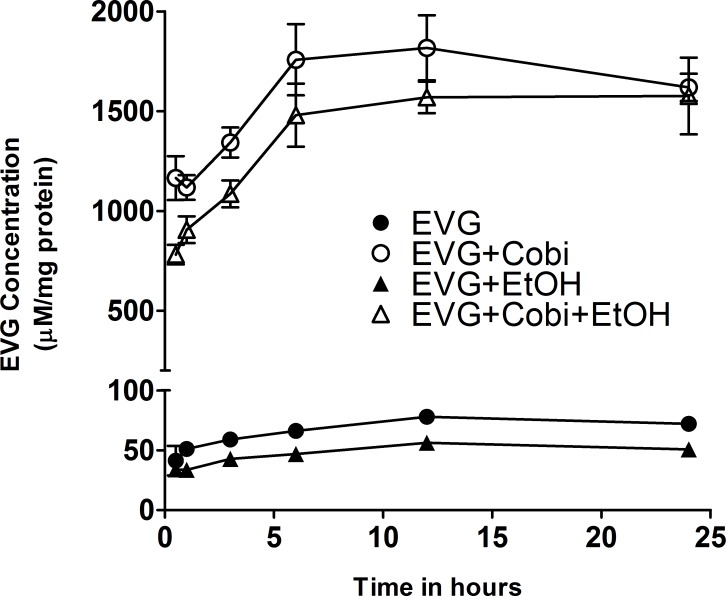
Concentration-time profiles for the accumulation of elvitegravir in U1 cells with or without cobicistat and ethanol. Results expressed as means ± SEM of the cellular concentrations measured (μM/mg of protein). EVG, Elvitegravir; COBI, Cobicistat; EtOH, ethanol.

The mean intracellular concentration-time curve for EVG, EVG co-exposed with COBI or ethanol, and EVG co-exposed with both COBI and ethanol are shown in Fig 1 and Table 1. The mean exposure of (AUC_tot_, μM*h/mg) of EVG alone (1733 ± 122) and EVG+COBI (40207 ± 3031) were decreased to 1733 ± 122 and 40207 ± 3031, respectively, when exposed along with ethanol. The difference is statistically significant (Table 1) and shows 39% (EVG vs EVG+EtOH t(4) = 2.794, p = 0.0491, student t test) and 26% (EVG+COBI vs EVG+COBI+EtOH t(7) = 3.413, p = 0.0112) decrease due to ethanol exposure. The maximal concentrations (C_max_) after treatment with EVG alone and EVG+COBI were 82 ± 4 μM and 1896 ± 162 μM, respectively. When cells were co-treated with EVG and EVG+COBI combinations along with ethanol, C_max_ was found to be 61 ± 5 μM and 1756 ± 53 μM, respectively. While ethanol decreased the maximal EVG concentrations (C_max_) by 26% (EVG vs EVG+EtOH t(5) = 3.587, p = 0.0158) after treatment with EVG alone, it had a lesser impact (7%) on EVG C_max_ when co-administered with COBI. The median T_max_ (hours; h) values for EVG and EVG along with COBI were found to be 12 h, and ethanol exposure demonstrated 6 h for EVG and 18 h for EVG+COBI exhibiting no significant influence of ethanol co-treatment on T_max_ of EVG.

**Table 1:** Pharmacokinetic parameters of elvitegravir in U1 cells. AUC_tot_, area under the plasma concentration-time curve from time 0 to 24 hours; C_max_, measured maximum plasma concentration; T_max_, time to reach peak concentration. *p< 0.05 compared to EVG, ^#^p<0.05 compared to EVG+COBI. Data expressed as mean ± SEM from 4–5 independent experiments for AUC_tot_ and C_max_, and as median (range) for T_max_. EVG, Elvitegravir; COBI, Cobicistat; EtOH, ethanol.

Pharmacokinetic parameters	EVG	EVG+COBI	EVG+EtOH	EVG+COBI+EtOH
AUC_tot_ (μMXh/mg)	1733 ± 122	40207 ± 1031	1055 ± 210*	34734 ± 1229^#^
C_max_ (μM)	82 ± 4	1896 ± 162	61 ± 5*	1756 ± 53
T_max_ (h)	12 (11.5)	12 (18)	18 (18)	6 (6)

### Ethanol and elvitegravir interaction on HIV-1 replication

**Fig 2:**
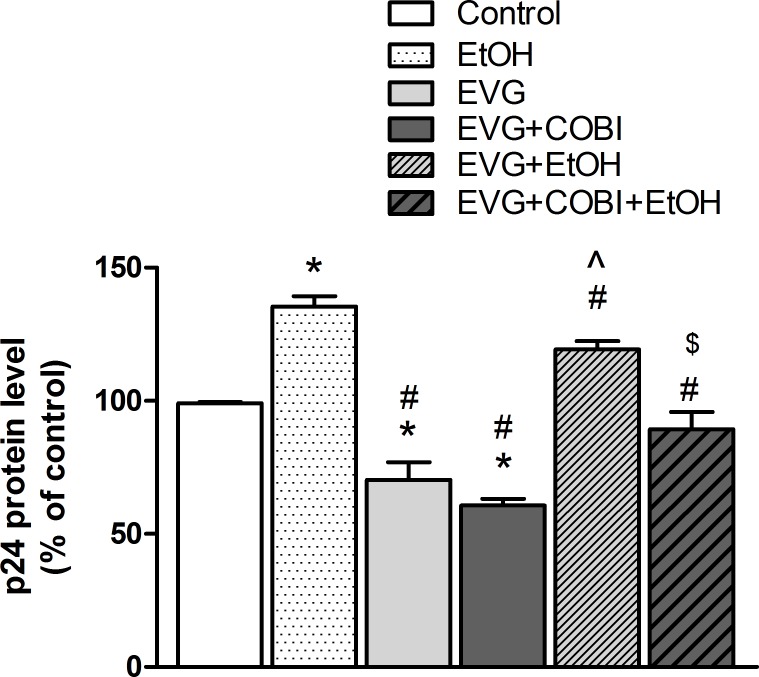
Alterations in HIV replication of U1 cells after drug exposure. PMA-differentiated U1 cells were treated with indicated drugs for 24 hours and HIV replication was determined using an ELISA for p24 levels in culture media. *p< 0.05 compared to control, ^#^p<0.05 compared to EtOH. ^p<0.05 compared to EVG, and ^$^p<0.05 compared to EVG+COBI. Values were mean ± SEM from 3 individual experiments. EVG, Elvitegravir; COBI, Cobicistat; EtOH, ethanol.

To understand the pharmacodynamic influence of ethanol and drug interaction, HIV-1 replication was indirectly assessed by measuring HIV p24 protein levels after treatment (24 hours). Data are presented in Fig 2. Ethanol exposure alone had a 35% increase compared to vehicle-treatment (F_(5,16)_ = 30.20, p = 0.0005, one-way ANOVA). Both EVG and EVG+COBI reduced the p24 levels by 30% and 40%, respectively, which is statistically significant compared to both vehicle (p = 0.0054 and p = 0.0003 respectively) and ethanol treatment groups (p<0.0001 and p<0.0001 respectively). Similarly, co-exposing cells to EVG and EVG+COBI along with ethanol enhanced p24 levels by 31% and 19%, respectively. This increase is statistically significant when compared to EVG (p< 0.0001) and EVG+COBI (p = 0.0055) groups but not with the vehicle treatment.

### Ethanol and EVG effects on CYP3A4, MRP1, and MDR1 protein expression

**Fig 3:**
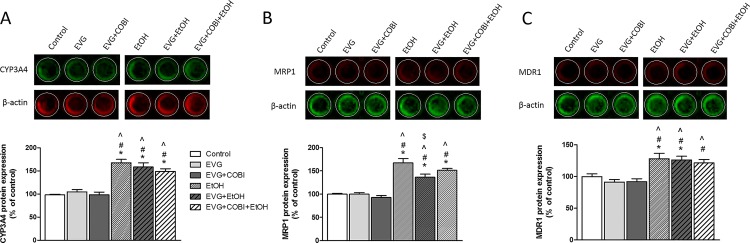
Induction of CYP3A4, MRP1, and MDR1 protein expression was analyzed by In-Cell Western (ICW) assay. Top section in each panel is a respective representative immunoblot of CYP3A4, MRP-1 or MDR1. Bottom plot in each panel demonstrates respective mean CYP3A4, MRP-1 or MDR1 fluorescence signal intensities of triplicates and error bars represent SEM values. *p< 0.05 compared to control, ^#^p<0.05 compared to EVG, and ^p<0.05 compared to EVG+COBI, ^$^p<0.05 compared to EtOH. Values were mean ± SEM from 3 individual experiments. EVG, Elvitegravir; COBI, Cobicistat; EtOH, ethanol.

In-Cell Western blot analysis was performed to understand the mechanism for alterations in intracellular PK parameters of EVG in response to ethanol exposure. CYP3A4, MRP1, and MDR1 proteins expression were detected, and the analyzed results are depicted in Fig 3, respectively. EVG alone or EVG treatment along with COBI showed no change in expression of CYP3A4; however, ethanol alone or in combination with EVG and EVG+COBI exhibited a significant induction of CYP3A4 compared to control (F_(5,51)_ = 26.73, p<0.0001, one-way ANOVA, Fig 3A). No significant difference was seen in ethanol exposed EVG or EVG+COBI combination from ethanol alone treatment.

Similarly, EVG or EVG+COBI treatments showed no effect on MRP1 expression. Ethanol upregulated MRP1 protein levels when treated alone or in combination with EVG or EVG+COBI compared to control (F_(5,50)_ = 28.85, p<0.001, one-way ANOVA, Fig 3B). EVG when exposed along with ethanol demonstrated an attenuation of ethanol-induced increase of MRP1 levels (p = 0.0043). Moreover, EVG exposure along with COBI and ethanol showed a decreasing trend in of ethanol-induced MRP1 expression but this was not statistically significant.

While drugs alone (EVG or EVG+COBI) showed no influence on the expression of MDR1, ethanol treatment both in the absence and presence of EVG exhibited a significant induction of MDR1 compared to control (F_(5,30)_ = 9.411, p<0.028, one-way ANOVA, Fig 3C). The impact of ethanol co-exposure with EVG and COBI was not statistically significant from control. No significant change was observed in EVG and EVG+COBI combinations that are co-treated with ethanol compared to ethanol alone exposure.

### Effect of MRP1 and MDR1 inhibition on intracellular EVG concentration

**Fig 4:**
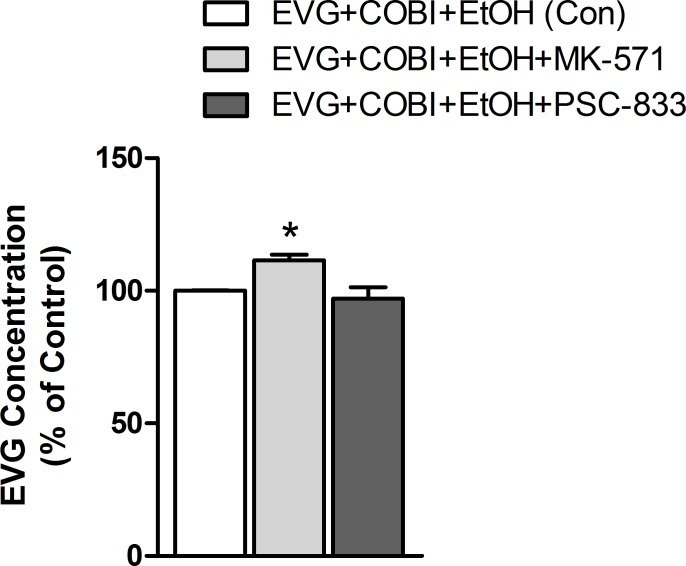
Intracellular elvitegravir concentration in the presence of MRP1 or MDR1 inhibitors and cobicistat + ethanol. EVG quantified from cell lysates using LC MS/MS after 24 hours treatment with indicated drugs. Data are expressed as mean ± SEM from 3 independent experiments. *p< 0.05 compared to EVG+COBI+EtOH (Control). EVG, Elvitegravir; COBI, Cobicistat; EtOH, ethanol.

Since the induction of CYP3A4, MRP1, and MDR1 induction in response to ethanol treatment (Fig 3), we tested the possibility of MRP1 and MDR1 transporter effects on EVG availability. For this experiment, ethanol co-treatment with EVG and COBI as control was chosen, which represents the influence of both ethanol and COBI. The cells that are exposed to the MRP1 inhibitor, MK-571 demonstrated a 12% increase in the EVG concentration compared to control and is statistically significant (F_(2,26)_ = 5.461, p = 0.031, one-way ANOVA, Fig 4). In contrary, the MDR1 inhibitor, PSC-833 showed no significant effect on intracellular EVG concentration.

### Effect of MRP1 and MDR1 inhibition in HIV replication

**Fig 5:**
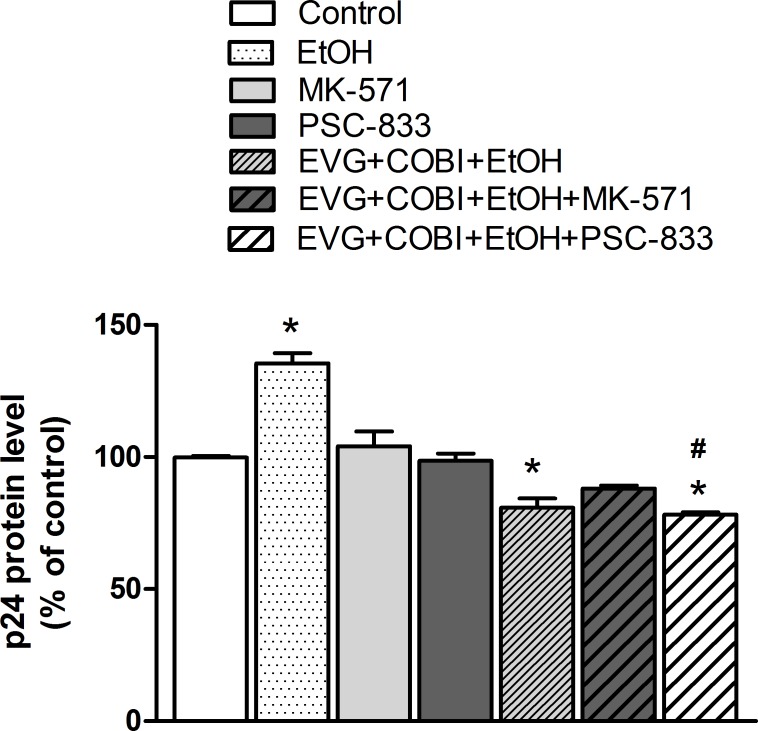
Changes in HIV replication of U1 cells after drug exposure. PMA-differentiated U1 cells were treated with indicated drugs for 24 hours and HIV replication was determined using an ELISA for p24 levels in culture media. *p< 0.05 compared to control, ^#^p<0.05 compared to PSC-833. Values were means ± SEM from 3 individual experiments. EVG, Elvitegravir; COBI, Cobicistat; EtOH, ethanol.

We determined HIV p24 levels in the presence of MK-571 or PSC-833 while keeping ethanol co-exposure with EVG and COBI as constant to observe the influence of these inhibitors on the HIV replication. As shown in Fig 5, ethanol increased the p24 levels by 36% compared to control (F_(6,20)_ = 18.89, p<0.0001, one-way ANOVA) but MK-571 or PSC-833 did not show any effect on the p24 levels. PSC-833 in combination with EVG, COBI, and ethanol showed a decrease in p24 levels compared to vehicle or PSC-833 alone control (p<0.05). However, both MK-571 and PSC-833 in the presence of EVG, COBI, and ethanol failed to show significant change when compared to EVG, COBI, and ethanol combination.

## Discussion

Optimum intracellular concentration is critical to the success of ART in HIV treatment and is affected by several external or internal dynamics. However, only a few clinical studies investigated the effects of ethanol on ART by measuring the plasma pharmacokinetics[21, 28, 29]. In general, it is conceived that small molecules that interact with ethanol dehydrogenase or CYP2E1 and are likely to be influenced by ethanol exposure. This interaction could lead to clinically significant changes in the pharmacokinetic properties of those drugs[30]. However, the influence of ethanol drinking on the intracellular concentration of ART or its effects on the pharmacodynamics is relatively unclear. Therefore, the current study investigated the intracellular drug-drug interactions between EVG and ethanol in combination with or without COBI in HIV-infected monocytic (U1) cells. Herein, we report a decreased EVG AUC and C_max_ values and increased HIV replication in the presence of ethanol. In addition, mechanistic studies revealed that ethanol induced alterations in EVG cellular pharmacokinetics and associated effect on HIV replication is, at least partially, due to induction of CYP3A4, MRP1, and MDR1 proteins.

EVG is required to penetrate the HIV-infected cell to exert its antiviral activity. Usually, ART plasma concentration is used to assess the intracellular drug level and to understand overall intracellular drug disposition[31] Currently, there is no information on EVG uptake into cells and the relationship between its plasma and cellular concentrations. Nonetheless, evaluation of therapeutic levels in the cells based on plasma concentrations may not be accurate especially for integrase inhibitors. Like nucleoside/nucleotide reverse transcriptase inhibitors, intracellular half-life of integrase inhibitors is critical to achieve antiviral effect[32]. For instance, an initial Phase III study of Isentress® (raltegravir), an integrase inhibitor show that 400 mg twice-daily is more effective than 800 mg once daily[8]. Moreover, considering high protein binding and poor cell penetration capability (~5% of plasma concentration) of integrase inhibitors[33, 34], small changes as a result of co-exposure of ethanol may greatly influence the intracellular concentration of EVG. Therefore, it is worth noting that the observed considerable influence of ethanol on the extent of EVG overall exposure irrespective of COBI presence may have impact on clinical outcomes in HIV treatment.

Our findings that ethanol causes an increase in HIV replication is in agreement with previously reported observations[35–37]. EVG or COBI-boosted EVG displayed a significant viricidal activity. However, this activity is not as pronounced as EVG effects in HIV-infected patients or on freshly infected cells[38, 39]. The inherent constitutive expression of HIV in the U1 cells could be the likely reason for this reduced effect of EVG in these experiments[40]. However, ethanol exposure increases HIV p24 levels despite the presence of EVG or EVG and COBI. This increase is attributable to either reduced intracellular drug exposure as described above or by stimulating HIV replication through other mechanisms. For example, a recent study showed that ethanol enhances HIV infection in cord blood monocyte-derived macrophages by inhibiting anti-HIV microRNA and interferon alpha expression[35]. This important finding will require further investigation using HIV-infected primary macrophages or clinical samples under both acute and chronic alcohol consumption conditions.

The intracellular concentration of drugs is majorly controlled by passive transport, active uptake, and efflux from the cells[31]. EVG is a substrate for CYP3A4, and this drug metabolizing enzyme is present in the monocytic cells[18, 41]. Ethanol alone or when co-administered with EVG and COBI upregulated CYP3A4 protein levels. This observation is consistent with the reported induction of CYP3A4 mRNA and protein in monocytes[18]. Similarly, recently we reported decreased microsomal CYP3A efficiency by ethanol in metabolizing EVG[25]. Of interest, other ART drugs such as PIs are also metabolized by CYP3A4. When purified CYP3A4 enzyme exposed to 20 mM ethanol and PIs at different concentrations, ethanol alters spectral binding as well as inhibitory properties of PIs with the enzyme[42]. In addition to modifying above properties ethanol also decreases the catalytic efficiency of the CYP3A4 to metabolize nelfinavir[43]. These effects of ethanol are in corroboration with other studies that used animal models and in vitro cell lines to demonstrate that in addition to CYP2E1 ethanol markedly influences CYP3A4 [44–46]. Taken together, these results suggest that ethanol can alter CYP3A4 mediated EVG metabolism by modulating CYP3A4 at different points including mRNA, protein or functional level.

Reported evidence indicates that ART intracellular levels are also dependent on MRP1 and MDR1 or other efflux transporters expressed in monocytes[47, 48]. Therefore, we tested the hypothesis that ethanol co-administration with EVG modulates these molecular targets, which in turn, influences intracellular level of EVG. Similarly, we have previously reported upregulation of MRP1 mRNA as a result of ethanol exposure to U937 monocytic cells[18]. Our current observations of induction of MRP1 by ethanol are in agreement with our earlier studies. Besides, a published study has demonstrated induction of CYP3A4 and MDR1 mRNA in human adenocarcinoma LS180 cells by EVG[49]. In contrary, we observed induction of these proteins by EVG only with the combination of ethanol but not by EVG alone. This discrepancy may arise due to comparison of mRNA expression with protein or use of different cell model systems in these studies. For instance, phenotypically different subsets of macrophages M1 and M2 exhibited significant variations in intracellular concentrations of lopinavir with elevated levels of MDR1 expression in M2 type[50]. Regardless, when cells were exposed to MRP1 and MDR1 inhibitors along with EVG, COBI, and ethanol combination, EVG intracellular concentration was slightly increased but no noticeable impact was observed on HIV replication. These results suggest that these efflux transporters may not be solely responsible for ethanol-mediated effects on intracellular EVG concentration.

In conclusion, our findings clearly suggest modulation of intracellular concentration of EVG and HIV-1 replication in HIV-infected U1 monocytic cells, perhaps through drug efflux transporter MRP1 and metabolic enzyme CYP3A4. The findings of this study could be strengthened by providing further evidence of the role of MRP1 and CYP3A4 using functional assays in HIV-infected primary macrophage. Antiretroviral drug-drug interactions can also be led by other CYPs and transporters and ethanol can induce other molecular targets[14] that may indirectly influence EVG concentration in the cells. Overall, the findings from this study suggest that ethanol-drug interactions may have clinically relevant effects on EVG containing ART regimens for HIV treatment. These important in vitro findings should guide the future in vivo or ex vivo drug-drug interaction studies to understand the effects of ethanol on intracellular concentration of this integrase inhibitor.

## Supporting information

S1 File Supporting information that contains original data and/or statistical analysis for Figs 1 to 5. (XLSX)
